# Supplementary material for: Resolving the full spectrum of human genome variation using Linked-Reads
Source: Genome Res. 2019 Apr;29(4):635–45. doi: 10.1101/gr.234443.118 (PMC6442396; doi:10.1101/gr.234443.118)
Supplement: Supplemental Material [file supp_gr.234443.118_Supplemental_longranger-2.2.zip › longranger-2.2.tar.bz2!/lr2.2-public/lib/stan/cmdstan/stan/doc/api/html/functions_vars_n.html]

Stan: Class Members - Variables


|  |  |
| --- | --- |
|  | Stan  2.5.0  probability, sampling & optimization |


- Main Page
- Related Pages
- Namespaces
- Classes
- Files

- Class List
- Class Index
- Class Hierarchy
- Class Members

- All
- Functions
- Variables
- Typedefs
- Enumerator
- Related Functions

- \_
- a
- b
- c
- d
- e
- f
- g
- h
- i
- k
- l
- m
- n
- o
- p
- q
- r
- s
- t
- u
- v
- w
- x
- y
- z


All Classes Namespaces Files Functions Variables Typedefs Enumerations Enumerator Friends Macros Pages

### - n -

- N\_
  : stan::agrad::LDLT\_alloc< R, C >
  , stan::gm::cholesky\_factor\_var\_decl
  , stan::gm::matrix\_var\_decl
  , stan::gm::row\_vector\_var\_decl
- n\_
  : stan::math::coupled\_ode\_observer
- N\_
  : stan::math::coupled\_ode\_system< F, double, double >
  , stan::math::coupled\_ode\_system< F, double, stan::agrad::var >
  , stan::math::coupled\_ode\_system< F, stan::agrad::var, double >
  , stan::math::coupled\_ode\_system< F, stan::agrad::var, stan::agrad::var >
  , stan::math::LDLT\_factor< T, R, C >
- n\_divergent\_
  : stan::mcmc::base\_nuts< M, P, H, I, BaseRNG >
- n\_leapfrog\_
  : stan::mcmc::base\_nuts< M, P, H, I, BaseRNG >
- n\_tree
  : stan::mcmc::nuts\_util
- name\_
  : stan::gm::arg\_decl
  , stan::gm::base\_var\_decl
  , stan::gm::fun
  , stan::gm::function\_decl\_def
  , stan::gm::variable
  , stan::gm::variable\_dims
  , stan::mcmc::base\_mcmc
- negated\_factor\_r
  : stan::gm::term\_grammar< Iterator >
- no\_op\_statement\_r
  : stan::gm::statement\_grammar< Iterator >
- nom\_epsilon\_
  : stan::mcmc::base\_hmc< M, P, H, I, BaseRNG >
- non\_lvalue\_assign\_r
  : stan::gm::statement\_grammar< Iterator >
- num\_dims\_
  : stan::gm::expr\_type
- num\_params\_r\_\_
  : stan::model::prob\_grad
- num\_samples
  : stan::io::stan\_csv\_metadata
- num\_warmup
  : stan::io::stan\_csv\_metadata
- num\_warmup\_
  : stan::mcmc::windowed\_adaptation
- nvaris
  : stan::agrad::OperandsAndPartials< T1, T2, T3, T4, T5, T6, T\_return\_type >


---

[ Stan Home Page ]

*© 2011–2014,
Stan Development Team.*
